# Mathematical modeling of the impact of Omicron variant on the COVID-19 situation in South Korea

**DOI:** 10.5808/gi.22025

**Published:** 2022-06-30

**Authors:** Jooha Oh, Catherine Apio, Taesung Park

**Affiliations:** 1 Department of Statistics, Seoul National University, Seoul 08826, Korea; 2 Interdisciplinary Programs in Bioinformatics, Seoul 08826, Korea

**Keywords:** COVID-19, mathematical models, Omicron, SARS-COV-2, variant

## Abstract

The rise of newer coronavirus disease 2019 (COVID-19) variants has brought a challenge to ending the spread of COVID-19. The variants have a different fatality, morbidity, and transmission rates and affect vaccine efficacy differently. Therefore, the impact of each new variant on the spread of COVID-19 is of interest to governments and scientists. Here, we proposed mathematical SEIQRDVP and SEIQRDV3P models to predict the impact of the Omicron variant on the spread of the COVID-19 situation in South Korea. SEIQEDVP considers one vaccine level at a time while SEIQRDV3P considers three vaccination levels (only one dose received, full doses received, and full doses + booster shots received) simultaneously. The Omicron variant’s effect was contemplated as a weighted sum of the delta and Omicron variants’ transmission rate and tuned using a hyperparameter k. Our models’ performances were compared with common models like SEIR, SEIQR, and SEIQRDVUP using the root mean square error (RMSE). SEIQRDV3P performed better than the SEIQRDVP model. Without consideration of the variant effect, we don’t see a rapid rise in COVID-19 cases and high RMSE values. But, with consideration of the Omicron variant, we predicted a continuous rapid rise in COVID-19 cases until maybe herd immunity is developed in the population. Also, the RMSE value for the SEIQRDV3P model decreased by 27.4%. Therefore, modeling the impact of any new risen variant is crucial in determining the trajectory of the spread of COVID-19 and determining policies to be implemented.

## Introduction

Coronavirus disease widely known as coronavirus disease 2019 (COVID-19) is a new disease caused by the severe acute respiratory syndrome coronavirus 2 (SARS-CoV-2) virus that emerged from Wuhan, China [1] and spread rapidly across the world becoming a global pandemic in March 2020 [2]. The pandemic caught many governments and people by surprise which led to the implementation of unprecedented intervention policies like school and workplace closures, suspension of public transportation, international travel restrictions, and so forth [3], with aims of mitigation and suppression. The reason was to not overwhelm the unprepared healthcare systems and lower the number of cases until a pharmacological solution was found [4,5]. In addition, many pharmaceutical companies in partnership with government bodies launched one of the fastest vaccine development projects of our decade leading to the development of multiple vaccines [6,7]. The Coalition for Epidemic Preparedness Innovations (CEPI) worked with global health authorities, governments, and vaccine developers to support the development of vaccines against COVID-19 [8].

This pandemic also attracted the interest of researchers from different fields since it was a new disease and its transmission pathways and fatality were not yet known. From the outbreak to 4 February, 2022, researchers have contributed a lot in forecasting, and understanding the transmission dynamics, the fatality of SARS-CoV-2, and the evolution of the pandemic, to help in the fight against this new global problem. Among the researchers, statisticians, epidemiologists, and mathematicians contributed to formulating models to capture the transmission dynamics of COVID-19 and forecasting the evolution of the pandemic among different populations amidst government interventions. These mainly included statistical models [9-18], deep-learning models [19-24] and mathematical models [1,14,25,26].

Statistical models offer more precise models and deep-learning techniques are the key to high-quality predictive models [27]. However, both statistical and deep-learning models require real data to make predictions. But with mathematical models, a set of mathematical equations that mimic the current situation is written, and solving them for certain parameters provide information about the disease characteristics [28]. Some of their advantages include mathematical models representing the real situation of the problem being solved and they do not require all data to be available for it to be fitted as deductions from known information about the situation can be used. Also, they can handle sudden changes and complexity with ease. Since the start of the COVID-19 pandemic, mathematical models have been at the forefront of determining and forecasting the spread of COVID-19 and shaping government policies around the world [28].

A seminal paper in 1927 introduced the Susceptible, Infectious, and Recovered (SIR), a mathematical model for infectious diseases [29]. Since then, with advances in information technology and fast computing methods, many variations of the SIR model have been developed. Because mathematical models can easily be understood and definite conclusions about the COVID-19 outbreak can be made from them, Susceptible, Exposed, Infectious and Recovered (SEIR), a modification of SIR and a cascade of other modifications have been constructed and developed for predicting COVID-19 since its declaration as a global pandemic [30-42].

Subsequently, on 8 December, 2020, 272 days after COVID-19 was declared a global pandemic, vaccination started in the United Kingdom [43]. Since then, as of 4 February, over 61.34% of the world population has received at least one dose of a COVID-19 vaccine, and 21.54 million doses of vaccines are administered daily around the world [44]. However, since then new variants of the SARS-CoV-2 virus have appeared. These variants have different transmissibility rates, fatality, and morbidity. Furthermore, existing vaccines have differing efficacy levels against these emerging variants [45,46]. Governments must make decisions and revise policies while considering these new developments like the impact of vaccinations and emerging variants on the spread of COVID-19. However, statistical and deep-learning models would require real data in substantial amounts to perform any forecasting or prediction. On the other hand, these new developments can easily be modeled with little or no data with mathematical models.

For the Korean COVID-19 situation, many models were employed to forecast the future COVID-19 situation in the country amidst government social distancing policies. One paper used the SIR model with time-dependent parameters and deep learning to forecast the spread of COVID-19 in South Korea [47]. Another analysis utilized the SIR model with breakpoint information that allows change in transmission rate at the breakpoints was established [48]. Other uses of the SIR model or its modification used for the Korean population are found elsewhere [49-53]. A modification of the SEIR model that considers transmission rates between age groups and vaccination was also formulated for the Korean population [54]. In this model, five additional groups; quarantined Q, unprotected U, vaccinated V, protected P, and deceased D were added to the standard SEIR model making it the SEIQRDVUP model.

Since the SARS-CoV-2 virus is an RNA virus and lacks the mismatch repair mechanism, the virus replication process is accompanied by a high mutation rate, hence the rise of variants [55]. Common mutant variants include B.1.1.7, B.1.351, B.1.1.28.1, B.1.617.2 (Delta), and B.1.1.529 (Omicron), which have all spread rapidly worldwide. The mutations make the virus more contagious (fast-spreading) and difficult to eliminate [56]. However, the SEIQRDVUP model and other previous methods cannot catch the sudden increase in daily cases caused by newer variants with higher transmission rates compared to a previously dominant variant.

To solve this limitation, we formulated a modification of the SEIQRDVUP model to consider a weighted sum of delta and Omicron variants’ transmission rates based on variants’ proportions together. In addition, three vaccination levels (only one dose received, full doses received, and full doses+booster shots received) were considered by adding three more compartments of vaccination (V1, V2, and V3) and the removal of the above-mentioned U compartment due to the use of a transmission rate that includes the effect of vaccine efficacy thereby eliminating the ineffectively vaccinated group, U. So, the Omicron variant’s effect was contemplated as a weighted sum of the delta and Omicron variants’ transmission rate. In this case, the Omicron variant’s transmission rate is assumed to be a multiple of the delta’s transmission rate, as explained in detail in the Methods section. This study aims to examine how the Omicron variant will affect the COVID-19 situation in Korea with our proposed SEIRQDVP and SEIRQDV3P models. The SEIQRDVP considers only one vaccination level at a time using only one vaccination compartment. However, the SEIQRDV3P considers all three vaccination levels simultaneously.

## Methods

### Proposed SEIQRDVP and SEIQRDV3P models

**Fig. 1.**
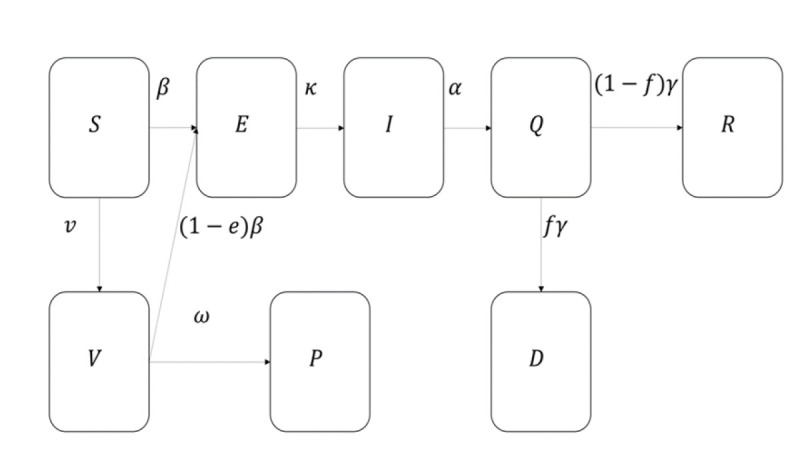
Flowchart of SEIQRDVP model.

Mathematical methods can be used for the prediction and forecasting of COVID-19 transmission [57-60]. Here, we proposed the SEIQRDVP model, and its flowchart is shown in Figure 1 below. The susceptible group S is the group of unvaccinated and uninfected people that can still be infected by the infectious group. The vaccinated group V is a group of people vaccinated and can still be infected by the infectious group but with a lower transmission rate. In this case, the efficacy of the vaccine is multiplied by the transmission rate. If a host in *S* or *V* group gets infected, this host becomes a host of the exposed group, *E*. After the incubation period, a host of *E* can infect *S* or *V* groups, which means that a host of *E* becomes a host of I, the infectious group. When a host of I is determined to be infected, a host will be isolated and becomes a host of an isolated group, Q, and loses the ability to infect others. An isolated host will be recovered or be dead and moves to group R or D, which are the recovered group and deceased group. Group P is the insusceptible group that has immunity. The following differential equations represent the SEIQRDVP model:


$$\frac{dS}{dt}=-\beta S\frac{I}{N}-v,$$

$$\frac{dE}{dt}=\beta \left(S+\left(1-e\right)V\right)\frac{I}{N}-\kappa E,$$

$$\frac{dI}{dt}=\kappa E-\alpha I,$$

$$\frac{dQ}{dt}=\alpha I-\gamma Q,$$

$$\frac{dR}{dt}=\left(1-f\right)\gamma Q,$$

$$\frac{dD}{dt}=f\gamma Q,$$

$$\frac{dV}{dt}=v-\left(1-e\right)\beta V\frac{I}{N}-\omega V,$$

$$\frac{dP}{dt}=\omega V,$$

N=S+E+I+Q+R+V+P.

where *β* is the transmission rate, *e* is vaccine efficacy, *f* is the mortality rate, *α,γ,k* and *w* are the duration periods from respective previous compartment to the next compartment, *N* is the total population, and *γ* is the isolation duration.

Previously determined model parameters from literature, κ,α,γ,f,e, and used in our analysis are listed in Table 1. We assumed that the vaccinated host gets immunity 42 days after their first vaccination which means that *1/ω* is assumed to be 42 [57]. In Fig. 1, *v* is provided by daily vaccinated cases. Consequently, the remaining parameter *β* is the only unknown parameter estimated by the least-squares method. This process is done using Runge-Kutta fourth-order method and the lsqcurvefit toolbox in MATLAB [61]. In addition, the daily cases are divided into segments with the breakpoints of these segments being determined from the changing levels of the stringency index due to changing government policies. The stringency index was obtained from the Oxford COVID-19 Government Response Tracker (OxCGRT) dataset from the Blavatnik School of Government and the University of Oxford [62,63]. β was estimated for each segment independent of other segments, therefore our proposed model included stringency index as a covariate.

**Fig. 2.**
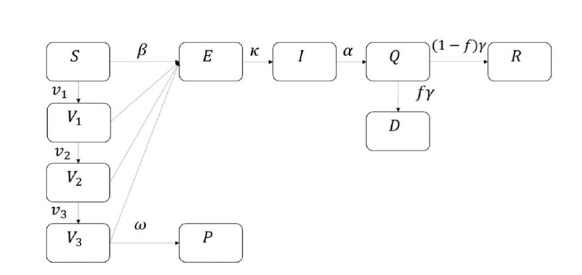
Flowchart of SEIQRDV3P model. Parameters for *V*_1_,*V*_2_,*V*_3_ to E are (1-*e*_1_)*β*, (1-*e*_2_)*β*,(1-*e*_3_)*β* like Fig. 1.

Moreover, the vaccination group can be divided into three: vaccinated (first vaccination), fully vaccinated (second vaccination), and boosted (third vaccination). We call the model that fits the three vaccination levels simultaneously, the SEIQRDV3P model, and its flowchart is shown in Fig. 2. In this model, *v*_1_,*v*_2_,*v*_3_ are provided by daily vaccinated, daily fully vaccinated, and daily boosted cases. Also, the efficacy of vaccination for each vaccination group is differently provided with *e*_1_, *e*_2_, *e*_3_ and values are 0.75, 0.80, 0.85 [69].

Lastly, the proportion of Omicron variants was reflected in the above model as a change in transmission rate, β. With a transmission rate of delta variant as β_D_ and transmission rate of Omicron variant as β_O_, we assumed that β_O_ is multiple of β_D_, which means β_O_=kβ_D_ with hyperparameter k. In our cases, we tried 1, 3, 5, and 7 as a value of the hyperparameter k, to track the recent rapid increase of the Omicron variant. The proportion of the Omicron variant in the population is modeled by the parameter w. The values of w lie between 0 and 1. The time-series variation of this parameter is known for both train and test data, but its variation for the coming days is unknown. So, a logistic function was fitted to predict the future behavior of w. Using past data on the proportion of the Omicron variant, the logistic function of the proportion of the Omicron variant against time was fitted by the least square method. Combining these results altogether, the final transmission rate became β_O_ w+β_D_ (1-w) which can be simplified as β_D_ {1+(k-1)w}. Since *k* and w are constants, the only parameter estimated is β_D_, and was estimated by the same method as the above models.

### Data

Information of daily cases, deaths, and the three vaccination levels used in the analysis was obtained from the *Our World in Data* website [70]. The daily recovered data is obtained from a web-based dashboard tracker of COVID-19 hosted by the Center for Systems Science and Engineering (CSSE) at Johns Hopkins University [71]. The proportion of cases because of the Omicron variant was gotten from GISAID, an Initiative dedicated to the tracking of virus variants from the influenza viruses and coronavirus [72,73]. This data was divided into train and test data. The training period was chosen from 20 September 2021 to 28 January 2022 since from this date (2021 September 20), the proportion of cases of delta variant had exceeded 90% of the cases. The test data period for prediction was from 29 January 2022 to 4 February 2022.

## Results

### SEIQRDVP and SEIQRDV3P models

Modifications of the basic SEIR model to the SEIQR model, to the SEIQRDVUP model, and then to our proposed SEIQRDVP and SEIQRDV3P models were done and the models' performances were compared. For each model, using train data, time-dependent β(t) using the different models were estimated by the least-squares method (LSE). SEIQR, SEIQRDVUP, and SEIQRDVP models’ results showed similar fitting with our proposed SEIQRDV3P model. We observed that except for the basic SEIR model and our SEIQRDV3P model, the other models had similar daily cases fitted curves.

Using test data, the prediction error of each model using actual confirmed cases and predicted confirmed cases from models was determined using root mean square error (RMSE). RMSE values for the SEIR, SEIQR, SEIQRDVUP, SEIQRDVP, and SEIQRDV3P models were calculated as 11,235, 5,079, 5,116, 5,115, and 5,101, respectively, as shown in Table 2. A general decrease in RMSE values with an increase in model complexity is observed. However, the difference in RMSE between SEIQR to SEIQRDV3P models is way smaller than the difference between SEIQR and SEIR models.

### Effect of Omicron variant

**Fig. 3.**
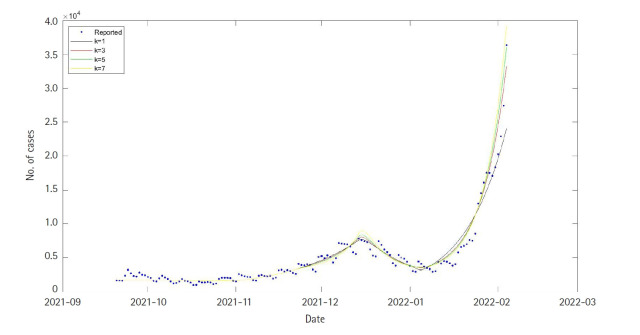
Fitted and predicted daily cases’ curves using the SEIQRDV3P model for different Omicron transmission rates *k* = 1,3,5,7. The time axis combines both train and test data periods, ranging is from 20 September 2021 to 4 February 2022.

From the above result, SEIQRDV3P and SEIQR models had the lowest but almost similar prediction errors, despite large differences in the model structure. This could be because of the recent Omicron situation in Korea. As transmissibility between the delta variant, which was originally dominant in Korea, and the Omicron variant which is now the dominant variant differ greatly. The SEIQRDV3P model which considers three vaccination levels simultaneously was updated to reflect the different effects of each variant due to their different transmissibility rate. Using train data, time-dependent *β_D_*(*t*) was estimated by the LSE method. In this case, hyperparameter *k* was chosen as 3, 5, 7, which means that the transmission rate of the Omicron is 3, 5, 7 times of transmission rate of the delta. For each selected *k*, the best fitted daily cases curves are shown in Fig. 3. The model with *k*=1 corresponds to the original SEIQRDV3P model. The x-axis (time) includes both the training and testing period.

**Fig. 4.**
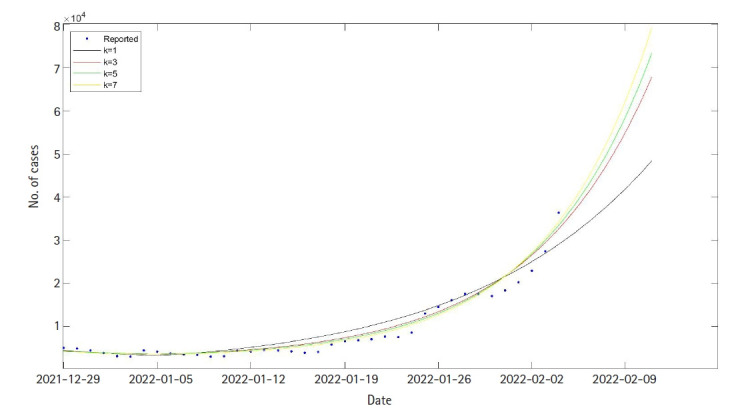
Forecasting of daily cases of Korea after test data, 5 February 2022 to 11 February 2022.

Also, RMSE values for each case were calculated as 5,101, 4,583, 4,200, and 3,705 for each value of hyperparameter k (1, 3, 5, and 7). As we include the effect of the Omicron variant in the SEIQRDV3P model, we can observe the dramatic decrease in RMSE values. Also, RMSE values decreased as the hyperparameter k increased, as shown in Table 2. This result implies that in a short period, the Omicron variant shows way larger transmissibility than the delta variant. Seven days’ prediction after the test data period, which is 5 February 2022 to 11 February 2022, is shown in Fig. 4.

## Discussion

Since the onset of the global COVID-19 pandemic, mathematical models have been at the forefront of forecasting the future pandemic situation hence policymaking by government bodies. Mathematical models are highly flexible and the impact of different scenarios on the transmission of COVID-19 can be incorporated and predicted, even with the unavailability of data. The mathematical compartmental SEIR model and many of its modifications have been developed.

Governments must revise their testing protocols, social distancing policies, and healthcare protocols with the emergence of each new variant, hence the need of modeling the impact of each emerging variant on the spread of COVID-19. Here, we proposed a modification of the published SEIQRDVUP model, the SEIQRDVP model which considers one vaccination group at a time, and the SEIQRDV3P model which models the 3 vaccination levels simultaneously and the impact of the Omicron variant. SEIQRDVP and SEIQRDV3P models’ performance were compared to SEIQRDVUP and other known compartmental mathematical models SEIR and SEIQR models. Firstly, without considering the Omicron variant rate, our SEIQRDV3P model doesn’t show much difference from other models contrasted here. This result implies that the SEIQRDV3P model cannot predict a rapid increase in daily COVID-19 cases without a previous increasing daily case pattern.

However, using a hyperparameter and a weighted sum of transmission rates between two variants, we were able to predict the rapid increase caused by the Omicron variant. Omicron rate considering weighted sum lowers the prediction error of the SEIQRDV3P model from 5,101.342 to 3,705.078 which is 27.4% less than the SEIQRDVUP model. Since the Omicron variant has a way larger transmission rate than delta or other previously known variants, it seems that daily incidences will keep increasing until herd immunity for the Omicron variant is formed in the population.

However, from January 2022, daily deaths, as well as severity, seem to have decreased considerably. This pattern can imply the low risk and mortality associated with the Omicron variant compared to the delta variant, or the impact of vaccination on the population. Therefore, before implementing the ‘Living with COVID-19’ policy in Korea [74], the prediction of deceased and serious patient cases should be preceded. This work can be done by developing the mortality rate in the SEIQRDV3P model to also consider the Omicron variant’s mortality with the weighted sum method.

Considering that each variant has its different transmissibility rates, fatality, impact on vaccine efficacy, and morbidity, this generates different model parameter values making it difficult to model all current variants in one model. Therefore, each variant would require its model. Currently, using different model parameters for each variant remained a limitation of this study which we try to solve in the future. Also, SARS-CoV-2 has been known to affect age groups differently. Furthermore, the impact of variant and vaccination policies across different age groups of the population will be considered in our future studies.

With the appearance of new COVID-19 variants appearing after a few months, the fight to end the spread of SARS-CoV-2 even with vaccination has been greatly challenged. These new variants have a different fatality, transmission rate, and efficacy from currently available vaccines. Therefore, their effect on daily cases, deaths, and implemented non-pharmacological policies is of interest to governments and scientists. With the proposed SEIQRDV3P model we found out the new Omicron variant will cause a rapid rise in COVID-19 cases in South Korea for some time until herd immunity is developed in the population.

## Figures and Tables

**Table 1.** Previously determined model parameters

Parameter	Description	Value
1/*κ*	The average duration from E to I	4.1 days [64-66]
1/*α*	The average duration from I to Q	6 days [64,65]
1/*γ*	The average duration from Q to R or D	20.1 days [67]
*f*	Mortality rate	0.09 [68]
*e*	Efficacy of vaccination	0.78 [69]

**Table 2.** RMSE values for each models and at different different *k* (SEIQRDV3P)

Model	RMSE	*k* SEIQRDV3P)	RMSE
SEIR	11,235.23	1	5,101.342
SEIQR	5,079.369	3	4,583.178
SEIQRDVUP	5,116.04	5	4,200.31
SEIQRDVP	5,115.755	7	3,705.078
SEIQRDV3P	5,101.342	-	-
RMS	-	-	-

RMSE, root mean square error.
